# A low-density SNP array for analyzing differential selection in freshwater and marine populations of threespine stickleback (*Gasterosteus aculeatus*)

**DOI:** 10.1186/1471-2164-15-867

**Published:** 2014-10-06

**Authors:** Anne-Laure Ferchaud, Susanne H Pedersen, Dorte Bekkevold, Jianbo Jian, Yongchao Niu, Michael M Hansen

**Affiliations:** Department of Bioscience, Aarhus University, Ny Munkegade 114, 8000 Aarhus C, Denmark; National Institute of Aquatic Resources, Technical University of Denmark, Vejlsøvej 39, 8600 Silkeborg, Denmark; BGI-shenzhen, Main Building, Beishan Industrial Zone, Yantian District, Shenzhen, 518083 China

**Keywords:** Threespine stickleback, Single nucleotide polymorphism, RAD sequencing, Low-density array

## Abstract

**Background:**

The threespine stickleback (*Gasterosteus aculeatus*) has become an important model species for studying both contemporary and parallel evolution. In particular, differential adaptation to freshwater and marine environments has led to high differentiation between freshwater and marine stickleback populations at the phenotypic trait of lateral plate morphology and the underlying candidate gene Ectodysplacin (EDA). Many studies have focused on this trait and candidate gene, although other genes involved in marine-freshwater adaptation may be equally important. In order to develop a resource for rapid and cost efficient analysis of genetic divergence between freshwater and marine sticklebacks, we generated a low-density SNP (Single Nucleotide Polymorphism) array encompassing markers of chromosome regions under putative directional selection, along with neutral markers for background.

**Results:**

RAD (Restriction site Associated DNA) sequencing of sixty individuals representing two freshwater and one marine population led to the identification of 33,993 SNP markers. Ninety-six of these were chosen for the low-density SNP array, among which 70 represented SNPs under putatively directional selection in freshwater *vs.* marine environments, whereas 26 SNPs were assumed to be neutral. Annotation of these regions revealed several genes that are candidates for affecting stickleback phenotypic variation, some of which have been observed in previous studies whereas others are new.

**Conclusions:**

We have developed a cost-efficient low-density SNP array that allows for rapid screening of polymorphisms in threespine stickleback. The array provides a valuable tool for analyzing adaptive divergence between freshwater and marine stickleback populations beyond the well-established candidate gene Ectodysplacin (EDA).

**Electronic supplementary material:**

The online version of this article (doi:10.1186/1471-2164-15-867) contains supplementary material, which is available to authorized users.

## Background

It is becoming increasingly evident that evolution is not just a long-term process on the scale of millennia; contemporary evolution can take place over just a few generations [1, 2]. Similarly, the importance of parallel evolution in populations facing similar environmental conditions and the role of gene reuse (or lack thereof) in this process is increasingly discussed [3–6]. The threespine stickleback (*Gasterosteus aculateus*) is distributed throughout the northern Hemisphere and shows extensive morphological and ecological variation [7]. Numerous resources, including its genome sequence are available, and the species has emerged as one of the most important models for studying both contemporary [8, 9] and parallel evolution [10–16]. Adaptation to freshwater and marine environments, respectively, has received particular attention due to the differences of plate morphology in the two environments and the finding of Ectodysplacin (EDA) as a candidate locus [10, 16, 17]. Nevertheless, other regions of the genome than that harboring EDA also show footprints of differential selection in freshwater and marine habitats [11–13], and in some cases these encompass non-coding and presumably regulatory regions [13].

In some geographical regions, notably Northern Europe, the patterns of divergence between marine and freshwater populations of threespine sticklebacks appear less distinct than in other regions, possibly reflecting gene flow overcoming selection [18, 19]. However, this has mainly been studied with specific focus on EDA and/or lateral plate morphology. Screening of adaptive divergence at other chromosomal regions could be achieved by whole genome sequencing or RAD (Restriction Associated DNA) sequencing [11, 13], though this precludes studies requiring large sample sizes. Also, a medium-density SNP chip has previously been constructed, encompassing 3,072 markers [12]. However in some situations, where analysis of many individuals from many localities is required, it would be preferable to invest more in sample size than in genomic resolution. This involves cases where hundreds of individuals are analyzed in order to assess e.g. temporal changes of allele frequencies as a result of selection, or hybrid zone dynamics [18–20]. Microsatellite loci have been developed that mark chromosomal regions under differential selection in freshwater and marine environments [16, 17], but development of a SNP array would allow for even faster and cost-efficient genotyping. In the present study, we therefore aimed at generating a low-density SNP array encompassing markers of chromosomal regions under differential freshwater-marine selection along with neutral markers for background, thus providing a resource for extensive studies of parallel evolution and marine-freshwater hybrid zone dynamics.

We identified SNPs based on RAD sequencing of one marine and two isolated freshwater populations. Based on these data we chose 96 SNPs for inclusion in the array. In order to validate the array we also analyzed a sample of threespine sticklebacks from a Danish river that represents a mixture of marine and freshwater morphs.

## Methods

### Ethical statement

Sampling of sticklebacks took place in accordance with Danish law and regulations. Threespine stickleback is not included in the Directive “Bekendtgørelse om fredning af visse dyre- og plantearter mv., indfangning af og handel med vildt og pleje af tilskadekommet vildt” (Directive on Protection of Certain Animal and Plant Species, Catch and Trade of Game, and Nursing of Wounded Game) by the Danish Ministry of the Environment. Catch of sticklebacks is therefore permitted unless it involves so high numbers of individuals that it would significantly affect the ecosystem, which was clearly not the case in this study. The fish were euthanized using an overdose of benzocaine and were subsequently stored in 96% ethanol.

### Sampled localities

**Figure 1:**
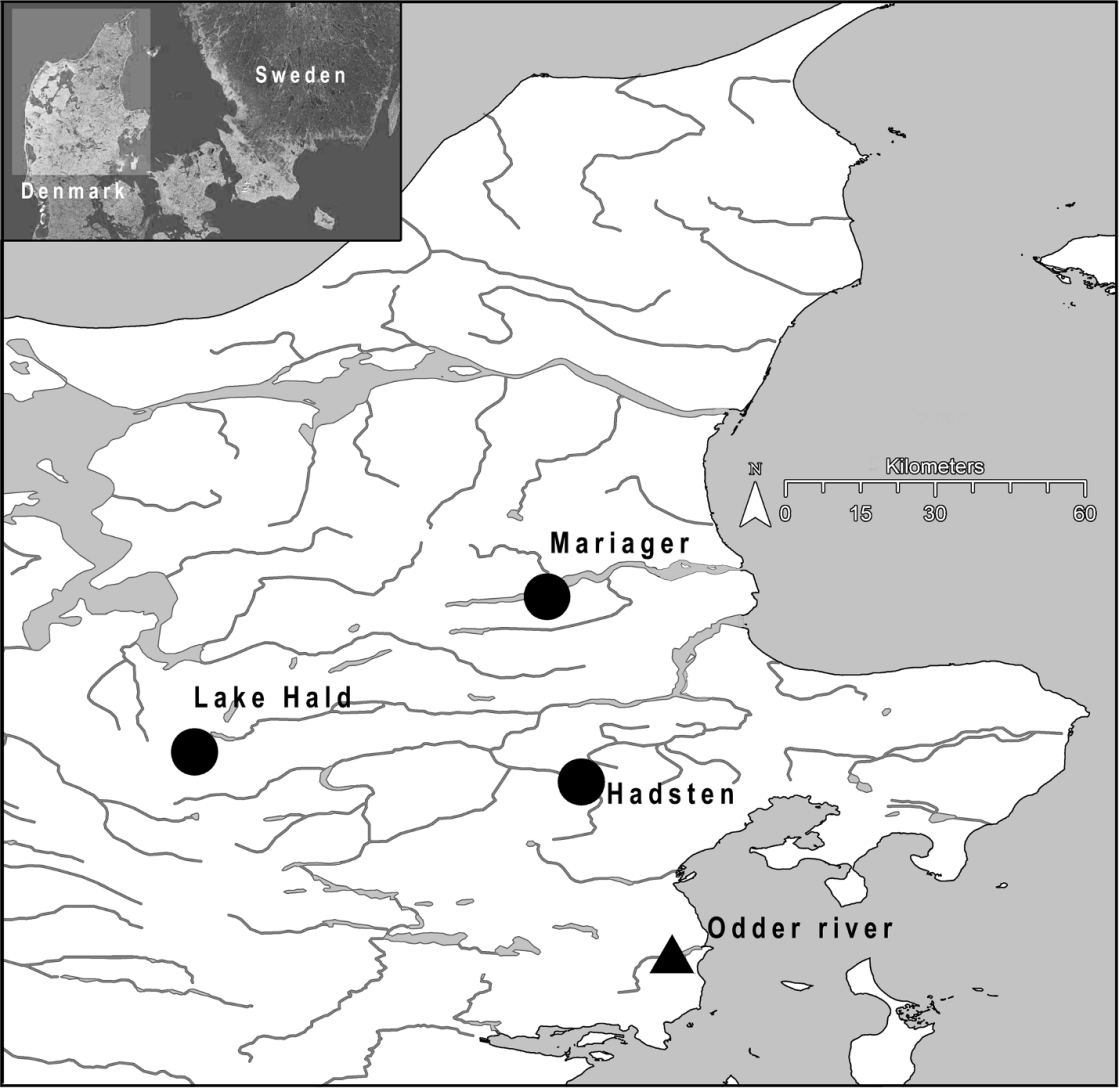
**Map showing the location of sampled three-spine stickleback populations in Jutland, Denmark.**

Sixty threespine sticklebacks, 20 from each site, were sampled by cast nets or minnow traps from three localities in Jutland, Denmark: 1) Lake Hald, a 3.3 km^2^ freshwater lake, 2) a small unnamed freshwater pond (ca. 0.01 km^2^) near the town of Hadsten and 3) the Mariager Fjord, a marine environment (see Figure 1). These individuals were analyzed using RAD sequencing [11, 21] in order to identify SNPs. 4) An additional 96 individuals were sampled close to the outlet of the Odder River, Jutland, Denmark (see Figure 1). Individuals from this estuarine population were genotyped in order to validate the generated SNP array. The first two samples (Lake Hald and Hadsten) consisted of morphs with low numbers of lateral plates (“low-plated”), as typically observed in freshwater [10]. The third, marine sample consisted of the typical marine morph with high numbers of lateral plates (“high-plated”), whereas the fourth estuarine population consisted of a mixture of low and high-plated morphs.

### RAD sequencing and SNP identification

Genomic DNA was extracted from muscle tissue using standard phenol-chloroform extraction. RAD sequencing was conducted by Beijing Genomics Institute (BGI, Hong Kong, China). The procedures for construction of libraries and Illumina HiSeq paired-end sequencing followed those described for European eel (*A. anguilla*) by Pujolar *et al.* [22], except for the fact that samples were digested with the restriction enzyme SbfI instead of EcoRI. Sequence lengths were 90 bp.

Only the first reads (with the restriction site) were used in subsequent analyses due to low coverage of the second reads (not containing the restriction site). The sequence reads were sorted according to their unique barcode tag and filtered and trimmed using the FASTX Toolkit (http://hannonlab.cshl.edu/fastx-toolkit). Final read lengths were trimmed to 75 nucleotides to avoid an increase of sequencing errors in the tail ends [22]. Reads of poor quality (with a Phred score < 10 per nucleotide position) were removed. Reads were subsequently aligned to the stickleback genome using Bowtie version 0.12.8 [23] with a maximum of 2 mismatches allowed between individual reads and the genome sequence. Alignments were suppressed for a particular read if more than one reportable alignment was present. This was done in order to minimize the occurrence of paralogous sequences in the data.

The reference-aligned data were subsequently used to identify SNPs and call genotypes. For this purpose we used the Refmap.pl pipeline in Stacks [24], implementing a maximum-likelihood model for SNP calling and filtering out RAD loci within individuals with a coverage < 10x. Furthermore, we required loci to be genotyped in at least 70% of the individuals from each population sample. Loci with a sequencing depth > 80x or exhibiting three alleles within individuals were also removed in order to avoid paralogs.

F_ST_ for each SNP between pairs of marine and freshwater populations was estimated using Populations implemented in Stacks [ [24] ]. The same pipeline was used for estimating sliding windows F_ST_ across 150,000 bp along each chromosome, based on a Gaussian Kernel smoothing function. Finally, the smoothed F_ST_ values were plotted using the R package [25].

### SNP low-density array design

Based on the outcome of the analysis of RAD data we selected 96 SNPs for inclusion in the low density SNP array. We selected SNPs 1) exhibiting high genetic differentiation between the two freshwater and marine populations, both at the individual SNPs and based on smoothed F_ST_ values, indicating possible diversifying selection; and 2) SNPs outside regions of elevated differentiation, presumably reflecting neutral markers. We used the threespine stickleback genome sequence to extend the flanking sequence to at least 100 bp to allow for optimal primer design. We also searched for possible candidate loci marked by the SNPs using the stickleback genome browser (http://sticklebrowser.stanford.edu), in which many genes are already annotated by name and putative orthology. The best Blast hit was used to assess the putative orthologous gene. The putative orthology relationships of the remaining genes, i.e. those that have not yet been annotated, were further analyzed by a Blast comparison of their predicted protein sequence against the NCBI protein database. The function of the candidate genes was assessed using two searchable databases: The *AmiGO 2* GO browser and an integrated database of human genes that also provides putative orthology with other vertebrates (http://www.genecards.org/).

The selected 96 SNPs were genotyped in 96 individuals from the Odder River population on 96.96 Dynamic Arrays (Fluidigm Corporation, SanFrancisco, CA, USA), using the Fluidigm EP1 instrumentation according to the manufacturer’s recommendations. The Fluidigm system uses nano-fluidic circuitry to simultaneously genotype up to 96 individuals at 96 loci (see [26] for a description of the Fluidigm system methodology). Genotypes were called using the Fluidigm SNP Genotyping Analysis software. We used Genalex 6.5 [27] to estimate expected and observed heterozygosity and test for Hardy-Weinberg equilibrium at each locus. Significance levels were adjusted using False Discovery Rate correction [28].

## Results

### RAD sequencing

RAD sequencing generated from 1.06 to 8.22 million reads per individual, with an average of 2.8 million reads. The mean depth of sequencing was 44.59. The number of reads retained through each step of the analysis is listed in Table 1. After all filtering steps in Stacks and post-filtering to remove possible paralogs, 19,793 loci were retained that represent 33,993 SNPs.

**Table 1:** **RAD sequencing statistics**

Population	N	Raw read count (M)	Read counts (M) after FASTX filtering and BOWTIE alignment	% Raw reads aligned	% Raw reads used
**Hadsten**	20	2.85	1.87	65.5	63.3
**Lake Hald**	20	2.80	1.12	40.0	38.8
**Mariager Fjord**	20	2.80	1.66	59.2	57.47

Summary-statistics for different steps of restriction-site associated DNA-sequencing (RAD-seq) data processing. N denotes the number of individuals in each sample. For each population the per individual average of raw read counts (Raw read count in Million bp), the number and percentage of high quality reads that were successfully aligned to the stickleback genome (Bowtie aligned), and the percentage of the aligned reads subsequently fed into Stacks (% Raw reads used) are presented.

**Figure 2:**
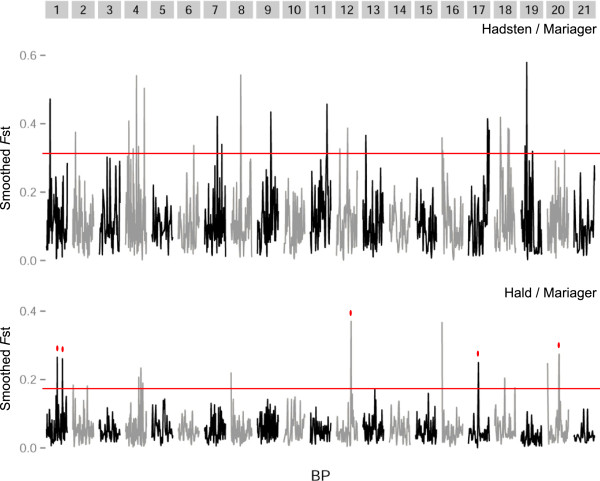
**Genome-wide distribution of smoothed F**_**ST**_ **estimates for pairwise comparisons between the Hadsten/Mariager and Lake Hald/Mariager populations.** Grey boxes indicate boundaries of chromosomes (from 1 to 21) and successive chromosomes are denoted by different shades of grey. Peaks above the red line correspond to the chromosomal regions exhibiting elevated differentiation that are referred to throughout the text, from which candidate SNPs for directional selection were chosen. Most peaks of elevated differentiation were shared between the two population comparisons, but in the cases where elevated differentiation was only observed in a single comparison this is denoted by a red dot.

Genome-wide F_ST_ was 0.056 between the Lake Hald (freshwater) and Mariager Fjord (marine) populations and 0.111 between Hadsten (freshwater) and the Mariager Fjord populations. Sliding window analysis of F_ST_ revealed high peaks of differentiation, potentially marking chromosome regions under differential selection in marine and freshwater. Twenty-one peaks distributed across 15 different chromosomes were thus identified in the Hadsten – Mariager Fjord comparison, whereas 15 peaks across 9 different chromosomes were revealed in the case of Hald – Mariager Fjord (Figure 2). Though most of these identified regions were found in both marine-freshwater population comparisons, some of them were found in only one of the two pairs.

### SNP low-density array design

We selected 96 SNPs for inclusion in the array. Twenty-six were chosen at random, but randomly distributed across 19 chromosomes to represent putatively neutral markers, with F_ST_ ranging from 0 to 0.18 between the two independent freshwater populations and the marine sample. The remaining 70 SNPs were chosen to reflect all of the high differentiation regions identified by the sliding-window approach. Some of the SNPs included represented high-differentiation peaks observed in both marine-freshwater population comparisons, but some were found to be outliers in only one of the two comparisons (Figure 2). The SNPs presumably under (hitchhiking) selection exhibited F_ST_ values ranging from 0.24 to 0.93 between Hadsten and Mariager Fjord and from 0.27 to 0.78 between Lake Hald and Mariager Fjord (Table 2). The number of outlier SNPs per chromosome ranged from 1 to 7. Considering all SNPs (neutral and under possible selection), each chromosome was represented by at least 4 SNPs.

**Table 2:** **List of the 96 selected SNPs**

SNP ID	Chr_position	p	q	F _ST_	Pop 1 ID	Pop 2 ID
5812b*	I_14574103	C	T	0.18	Hadsten	Mariager
27027*	II_13068160	A	G	0.00	Hadsten	Mariager
1800*	II_19830429	G	T	0.12	Hadsten	Mariager
1139*	III_9900776	C	T	0.03	Hadsten	Mariager
2620*	IV_10499598	C	T	0.07	Hadsten	Mariager
11120*	V_10474117	C	T	0.14	Hadsten	Mariager
9990*	VI_10404859	C	T	0.06	Hadsten	Mariager
10561*	VI_2863536	A	G	0.01	Hadsten	Mariager
9202*	VII_22894561	A	G	-0.12	Hadsten	Mariager
35752*	VII_4229364	A	G	0.05	Hadsten	Mariager
7275*	VIII_11705544	C	T	0.00	Hadsten	Mariager
28703*	VIII_14145458	A	C	-0.05	Hadsten	Mariager
4390*	IX_10719826	A	G	-0.04	Hadsten	Mariager
16548*	XI_11232372	G	T	-0.01	Hadsten	Mariager
13177*	XII_10200557	C	T	-0.11	Hadsten	Mariager
14236*	XIV_10623162	A	G	0.04	Hadsten	Mariager
15044*	XIV_7617082	C	T	-0.07	Hadsten	Mariager
20574*	XV_10990137	A	G	-0.01	Hadsten	Mariager
20825*	XV_14641595	C	T	-0.01	Hadsten	Mariager
31954*	XVII_1792778	A	T	-0.03	Hadsten	Mariager
15728*	XIX_1761203	C	G	-0.01	Hadsten	Mariager
22319*	XX_12299466	A	G	0.18	Hadsten	Mariager
32977*	XX_15123750	A	C	-0.04	Hadsten	Mariager
21643*	XXI_10893532	C	G	0.05	Hadsten	Mariager
21858*	XXI_4924010	A	G	-0.10	Hadsten	Mariager
33523*	Scaffold_122_287328	G	T	0.09	Hadsten	Mariager
5812	I_14574107	C	T	0.50	Hald	Mariager
5939	I_16513837	C	T	0.57	Hadsten	Mariager
28321	I_21607623	C	T	0.27	Hald	Mariager
28526	I_4931967	A	C	0.89	Hadsten	Mariager
6844	I_4932075	A	T	0.85	Hadsten	Mariager
1955	II_22061028	G	T	0.81	Hadsten	Mariager
2113	II_3125972	A	C	0.80	Hadsten	Mariager
2114	II_3182440	A	G	0.72	Hadsten	Mariager
276	III_13446716	A	G	0.67	Hadsten	Mariager
3231	IV_20387384	T	C	0.71	Hadsten	Mariager
3644	IV_29334535	A	G	0.70	Hadsten	Mariager
27608	IV_29334612	A	T	0.78	Hadsten	Mariager
3851	IV_3216905	A	T	0.70	Hadsten	Mariager
4073	IV_5292424	A	C	0.40	Hadsten	Mariager
27768	IV_6660266	A	T	0.77	Hadsten	Mariager
27791	IV_8128962	G	T	0.70	Hadsten	Mariager
29903	V_8795372	A	G	0.65	Hadsten	Mariager
10131	VI_12603660	G	T	0.68	Hadsten	Mariager
8466	VII_11202861	A	G	0.61	Hadsten	Mariager
9026	VII_19985290	C	G	0.79	Hadsten	Mariager
9206	VII_22946194	A	G	0.91	Hadsten	Mariager
7745	VIII_17638021	C	T	0.82	Hadsten	Mariager
7807	VIII_18320215	A	G	0.67	Hadsten	Mariager
7808	VIII_18320304	A	G	0.76	Hadsten	Mariager
8351	VIII_9101385	A	G	0.52	Hadsten	Mariager
4521	IX_13007542	G	T	0.40	Hadsten	Mariager
5200	IX_5019009	C	T	0.78	Hadsten	Mariager
5238	IX_5337004	C	G	0.77	Hadsten	Mariager
23341	X_11668366	C	G	0.36	Hadsten	Mariager
33387	X_6967185	A	C	0.26	Hadsten	Mariager
16649	XI_12761116	C	T	0.69	Hadsten	Mariager
16691	XI_13340957	C	T	0.70	Hadsten	Mariager
31479	XI_9810247	C	T	0.50	Hadsten	Mariager
13340	XII_12843029	A	G	0.78	Hald	Mariager
13682	XII_18204005	C	T	0.68	Hadsten	Mariager
13744	XII_3061560	A	G	0.60	Hadsten	Mariager
30542	XII_8981405	C	T	0.39	Hadsten	Mariager
14188	XII_9924630	A	G	0.54	Hadsten	Mariager
11996	XIII_11719547	C	T	0.47	Hadsten	Mariager
19976	XVI_18491	C	T	0.47	Hadsten	Mariager
20063	XVI_3012006	C	T	0.24	Hadsten	Mariager
35236	XVI_5021761	C	T	0.54	Hadsten	Mariager
20222	XVI_565127	C	T	0.54	Hadsten	Mariager
20238	XVI_593641	C	T	0.89	Hadsten	Mariager
18651	XVII_11584572	C	T	0.68	Hadsten	Mariager
18814	XVII_13715805	A	G	0.70	Hadsten	Mariager
32060	XVII_7058248	C	T	0.49	Hald	Mariager
17506	XVIII_10340214	A	G	0.63	Hadsten	Mariager
17577	XVIII_11202384	G	T	0.49	Hadsten	Mariager
17787	XVIII_14143185	A	C	0.76	Hadsten	Mariager
18047	XVIII_3081169	A	T	0.26	Hadsten	Mariager
18262	XVIII_6321841	C	T	0.77	Hadsten	Mariager
15358	XIX_11929930	A	G	0.62	Hadsten	Mariager
30997	XIX_16674218	A	T	0.69	Hadsten	Mariager
30997b	XIX_16674221	C	G	0.68	Hadsten	Mariager
15971	XIX_3850285	G	T	0.48	Hadsten	Mariager
31101	XIX_5560633	C	T	0.72	Hadsten	Mariager
22229	XX_10890902	A	G	0.50	Hald	Mariager
32994	XX_15998772	C	T	0.53	Hadsten	Mariager
22693	XX_17936432	A	G	0.93	Hadsten	Mariager
23102	XX_7553459	A	C	0.56	Hadsten	Mariager
21688	XXI_11538922	C	T	0.65	Hadsten	Mariager
21693	XXI_11580802	A	G	0.69	Hadsten	Mariager
22037	XXI_8170313	A	T	0.71	Hadsten	Mariager
24457	Scaffold_122_232322	A	G	0.83	Hadsten	Mariager
25425	Scaffold_27_3893488	C	T	0.59	Hadsten	Mariager
33942	Scaffold_309_4735	A	G	0.51	Hadsten	Mariager
26062	Scaffold_58_401854	A	C	0.57	Hadsten	Mariager
26071	Scaffold_58_511232	C	T	0.45	Hadsten	Mariager
26405	Scaffold_76_220535	C	T	0.59	Hadsten	Mariager

The 26 putatively neutral SNPs are indicated by asterisks (*) following the SNP IDs. Chr_position denotes the position of the SNPs in the threespine stickleback genome [13]. p and q are the two alleles found at the SNP position. F_ST_ denotes differentiation at the SNPs between population 1 and population 2.

The potential candidate loci for the SNPs under selection, along with their ontological relationships (when available) are listed in Table 3. This table lists 71 candidate genes identified from 20 chromosomes, 7 of which are involved in functions related to morphogenesis and growth, 2 related to skeletal biology, 5 related to kidney functions and 11 involved in osmoregulation. The remaining 46 candidate genes are associated to other functional categories, such as immune response, hormonal system or vision (see Table 3 for details). We chose not to include SNPs close to EDA, as this gene is usually analyzed using an indel (insertion-deletion) marker (Stn381) that is not suitable for inclusion in the array [10, 18]. Among the SNPs included in the array, the one closest to the EDA gene is situated more than 2.3 Mb away and therefore not showing tight linkage relationships. All sequences along with SNP positions used for generating the array are listed in Additional file 1: Table S1.

**Table 3:** **Identified candidate genes for freshwater** ***vs.*** **marine adaptation in threespine stickleback**

SNP ID	Chr_position	F _ST_	Candidate gene	Related function	MG	SB	KF	OM	OF
5812	I_14574107	0,5	Teneurin transmembrane protein 1 (ODZ1 )	morphogenesis	**×**				
5939	I_16513837	0,57	Claudin 4 (CLDN4)	internal organ development	**×**				
28321	I_21607623	0,27	insulin-like growth factor binding protein 2 (IGFBP2)*	growth and developmental rates	**×**				
28526	I_4931967	0,89	maltase-glucoamylase (alpha-glucosidase) (MGAM)	digestion					**×**
6844	I_4932075	0,85	maltase-glucoamylase (alpha-glucosidase) (MGAM)	digestion					**×**
1955	II_22061028	0,81	microfibrillar-associated protein 1 (MFAP1)	elastik fibres and collagen formation				**×**	
2113	II_3125972	0,8	ADAM metallopeptidase with thrombospondin type 1 motif, 18 (ADAMTS18)*	tumor supressor, eye development					**×**
2114	II_3182440	0,72	testis-specific serine kinase 3 (TSSK3)*	germ cell development, protein kinase activity					**×**
276	III_13446716	0,67	RUN and SH3 domain containing 1 (RUSC1)*	neuronal differentiation, cytoplasmic development	**×**				**×**
3231	IV_20387384	0,71	family with sequence similarity 19 (chemokine (C-C motif)-like) (FAM19A1)	regulators of immune and nervous cells					**×**
3644	IV_29334535	0,7	coiled-coil-helix-coiled-coil-helix domain containing 3 (CHCHD3)	crista integrity and mitochondrial function					**×**
27608	IV_29334612	0,78	coiled-coil-helix-coiled-coil-helix domain containing 3 (CHCHD3)	crista integrity and mitochondrial function					**×**
3851	IV_3216905	0,7	polycomb group ring finger 1 (PCGF1)	early embryonic development	**×**				
4073	IV_5292424	0,4	vascular endothelial growth factor B (VEGFB)	vascular endothelial growth					**×**
27768	IV_6660266	0,77	family with sequence similarity 70, member A (FAM70A)	transmembrane protein				**×**	
27791	IV_8128962	0,7	heparan sulfate (glucosamine) 3-O-sulfotransferase 1 (HS3ST1)	synthesis of anticoagulant					**×**
29903	V_8795372	0,65	retinol binding protein 4, plasma (RBP4)*	cardiac regulation, kidney filtration, retinal binding			**×**		**×**
10131	VI_12603660	0,68	glutamate receptor, ionotropic, delta 1 (GRID1)	nervous system					**×**
8466	VII_11202861	0,61	lens intrinsic membrane protein 2 (LIM2)	eye development and cataractogenesis					**×**
9026	VII_19985290	0,79	SCC-112	immune responses					**×**
9206	VII_22946194	0,91	RAD50	cell growth and viability					**×**
7745	VIII_17638021	0,82	tumor protein p63 (TP73L)	regulation of epithelial morphogenesis					**×**
7807	VIII_18320215	0,67	WW and C2 domain containing 1 (Wwc2)*	memory performance, regulation of organ growth	**×**				**×**
7808	VIII_18320304	0,76	WW and C2 domain containing 1 (Wwc2)*	memory performance, regulation of organ growth	**×**				**×**
8351	VIII_9101385	0,52	nephrosis 2, idiopathic, steroid-resistant (NPHS2)*	renal regulation, cell development	**×**		**×**		
4521	IX_13007542	0,4	phosphatidylinositol transfer protein, cytoplasmic 1 (PITPNC1)*	cell signaling and lipid metabolism					**×**
5200	IX_5019009	0,78	retinoblastoma binding protein 6 (RBBP6)*	suppresses cellular proliferation, embryonic development					**×**
5238	IX_5337004	0,77	KIAA0922	immune responses					**×**
23341	X_11668366	0,36	protein kinase (cAMP-dependent, catalytic) inhibitor beta (PKIB)	urinary regulation			**×**		
33387	X_6967185	0,26	Transcription factor EF1 (EF1)	regulates dendritic spine morphogenesis		**×**			
16649	XI_12761116	0,69	ATPase, Ca++ transporting, cardiac muscle, slow twitch 2 (ATP2A2)*	contraction/relaxation muscle cycle, heart regulation					**×**
16691	XI_13340957	0,7	transcription elongation factor B (SIII), polypeptide 2 (TCEB2)	renal regulation			**×**		
31479	XI_9810247	0,5	ATP-binding cassette, sub-family A (ABC1), member 3 (ABCA3)*	programmed cell death, membrane regulation		**×**			**×**
13340	XII_12843029	0,78	COMM domain containing 7 (COMMD7)	hepato cellular growth					**×**
13682	XII_18204005	0,68	TPX2, microtubule-associated (TPX2)	cell development					**×**
13744	XII_3061560	0,6	keratin 18 (KRT18)	internal organ development	**×**				
30542	XII_8981405	0,39	erythrocyte membrane protein band 4.1-like 1 (EPB41L1)*	neuronal plasma regulation, cytoskeleton regulation				**×**	**×**
14188	XII_9924630	0,54	suppression of tumorigenicity 5 (ST5)	immune responses					**×**
11996	XIII_11719547	0,47	transient receptor potential cation channel, subfamily M, member 3 (TRPM3)	mediates calcium entry				**×**	
19976	XVI_18491	0,47	MMADHC (uc010fnu.1) (CR595331)	vitamine B12 metabolism				**×**	
20063	XVI_3012006	0,24	sodium leak channel, non-selective (VGCNL1)	neuronal background sodium leak conductance, cell death					**×**
35236	XVI_5021761	0,54	retinoid X receptor, alpha (RXRA)*	retinoid development, heart development and morphogenesis	**×**				**×**
20222	XVI_565127	0,54	FLJ10154	hormonal expression					**×**
20238	XVI_593641	0,89	FLJ10154	hormonal expression					**×**
18651	XVII_11584572	0,68	EPH receptor A8 (EPHA8)	nervous system development					**×**
18814	XVII_13715805	0,7	PDZ domain containing ring finger 3 (PDZRN3)	myogenic differentiation	**×**				
32060	XVII_7058248	0,49	vitamin D (1,25- dihydroxyvitamin D3) receptor (VDR)	hormone receptor for vitamine D3, related to bone density		**×**			
17506	XVIII_10340214	0,63	FBJ osteosarcoma oncogene (FOS)	cell proliferation, differentiation, transformation					**×**
17577	XVIII_11202384	0,49	iodotyrosine deiodinase (C6orf71)	thyroid hormone production					**×**
17787	XVIII_14143185	0,76	phospholipase C, beta 1 (PLCB1)	intracellular transduction				**×**	
18047	XVIII_3081169	0,26	regulatory factor X, 6 (RFXDC1)	Production of insulin					**×**
18262	XVIII_6321841	0,77	estrogen receptor 2 (ER beta) (ESR2)	hormonal receptor, gametogenesis					**×**
15358	XIX_11929930	0,62	death-associated protein kinase (DAPK2)	programmed cell death					**×**
30997	XIX_16674218	0,69	lipase maturation factor 2 (LMF2)	maturation of the endoplasmic reticulum				**×**	
30997b	XIX_16674221	0,68	lipase maturation factor 2 (LMF2)	maturation of the endoplasmic reticulum				**×**	
15971	XIX_3850285	0,48	Fc receptor-like A (FCRLM1)	immune responses					**×**
31101	XIX_5560633	0,72	AK130540	salivary gland					**×**
22229	XX_10890902	0,5	cornifelin (CNFN)	ion transport across squamous epithelia, keratinization				**×**	
32994	XX_15998772	0,53	ubiquilin 4 (UBQLN4)	proteasomal protein degradation					**×**
22693	XX_17936432	0,93	TAF12 RNA polymerase II, TATA box binding protein (TBP)-associated factor (TAF12)	transcriptional activators					**×**
23102	XX_7553459	0,56	metastasis suppressor 1 (MTSS1)	metastases supressor					**×**
21688	XXI_11538922	0,65	AK095260	osmoregulation				**×**	
21693	XXI_11580802	0,69	cadherin 20 (CDH20)	tumor suppressor					**×**

Candidate genes are identified for 63 SNPs under putative directional selection. Note that 7 out of the 70 putative outliers SNPs were not found near to a coding gene and are not reported in the table. This concerns the six SNPs identified in diverse scaffold and one SNP (22037) in chromosome XX (see Table 2). Genes are assigned to one of the following categories: MG = Morphogenesis and Growth, OM = Osmoregulation, SB = skeletal Biology, KF = Kidney Function, OF = Other Function. These putative functions have been assessed using both the GeneCard database (http://www.genecards.org/) and *AmiGO 2* GO browser. In cases of multiple functions assigned to a single gene, this is denoted by “*”. For genes with multiple functions, only main functions previously documented in vertebrate species are reported.

Validation of the array based on analysis of 96 individuals from the Odder River provided results for all SNPs. However, there was significant drop-out at the markers 19976 and 26062 indicating technical problems with these two SNPs. Seven loci showed low expected heterozygosity (H_e_ < 0.05), whereas mean H_e_ across all loci was 0.226 (Additional file 2: Table S2). Twelve loci showed deviations from Hardy-Weinberg equilibrium, possibly reflecting that samples were taken in a mixture zone between freshwater and marine sticklebacks (Additional file 2: Table S2). Genotypic data for all SNPs and individuals are provided in Genalex 6.5 [27] format in Additional file 3.

## Discussion

### Development and utility of low density SNP chips

We are currently witnessing a transition from population genetics to population genomics, particularly mediated by the development of Next Generation Sequencing [29–31]. Whereas this allows for addressing research questions at the level of entire genomes [13, 32, 33], the methods used also provide resources that can be used for generating markers for more specific purposes. For instance, Hess et al. [34] conducted a population genomics study of Pacific lampreys using RAD sequencing, and subsequently used RAD data to construct a 96 SNP chip including markers that could be used for species identification, for general studies of genetic population structure and for screening loci previously suggested to be under directional selection [35]. Similarly, Pujolar *et al.* [36] used RAD sequencing of European (*Anguilla anguilla*) and American eel (*A. rostrata*) to develop a 96 SNP chip encompassing markers diagnostic for the two species. This resource was subsequently used for tracing hybridization between the two species several generations back in time.

The SNP chip developed in the current study similarly distills information derived from RAD sequencing. The 96 SNPs encompass markers of chromosomal regions that exhibit elevated differentiation in comparisons involving a marine population and two independent freshwater stickleback populations, possibly reflecting diversifying selection. It therefore provides a useful resource for analyzing differential adaptive responses in freshwater and marine sticklebacks and the extent to which this reflects parallel evolution. Nevertheless, it also involves some important caveats. First, although there is evidence for geographically widespread parallel evolution and gene reuse when marine sticklebacks colonize freshwater environments [11, 13, 16], there are clearly also examples of non-parallel adaptive responses [16], either reflecting differences in local freshwater environments or different genetic architecture underlying similar phenotypes. Our inclusion of SNPs therefore undoubtedly represents some degree of ascertainment bias [37, 38], particularly in terms of not identifying chromosomal regions under selection in other freshwater populations than those used for identifying SNPs. Second, three-spine stickleback is widespread across the Northern Hemisphere, and there is presumably a geographical limit defined by phylogeographical relationships beyond which many of the SNPs are no longer polymorphic; this can be regarded as another aspect of ascertainment bias. The developed SNP chip may therefore be of primary use in North-Western Europe, encompassing the North Sea and Baltic Sea regions.

Other marker resources have been developed for three-spine stickleback, including a 3,072 SNP chip [12], a resource of 158 microsatellite markers linked to physiologically important genes [17] and a resource of 110 SNPs representing both genic and non-genic regions [39]. Compared to the 3,072 SNP chip [12], the array developed in the present study obviously provides less dense genome coverage, but is also cheaper in running costs and specifically targeted towards freshwater-saltwater adaptation. Compared to the microsatellite resource [17], our 96 SNP array provides faster genotyping. On the other side, marker-by-marker multiallelic microsatellites provide more statistical power than diallelic SNPs [39–41]. A further important difference between 1) the microsatellite resource [17] and the 110 SNP resource [39] on the one side and 2) the current 96 SNP array on the other side consists in the choice of markers. Microsatellites and approximately half of the 110 SNPs were chosen based on the criterion that they should be linked to physiologically important genes [17]. In contrast, 70 of the SNPs included in the 96 SNP array were chosen from genomic regions exhibiting elevated differentiation, regardless of their linkage to candidate genes. There is increasing evidence that non-coding DNA may be of functional importance and potentially under selection [13, 42–44]. Indeed, 7 of the 70 SNPs under putative directional selection could not be linked to a candidate gene and could therefore potentially mark regulatory regions under selection. In total, our resource can be considered unbiased with respect to prior choice of candidate genes, but can be subject to ascertainment bias given that markers were chosen based on genetic differentiation between a subset of freshwater and marine populations. On the other side, the microsatellite resource by Shimada *et al.* [17] and a major part of the SNP resource by DeFaveri *et al*. [39] are specifically targeted towards genes of physiological importance but do not involve ascertainment bias in terms of choosing loci exhibiting high differentiation. Hence, there are pros and cons with both approaches and the choice of markers and methods may depend on the specific study and research question.

### Candidate genes for marine and freshwater adaptation

Similar to previous studies undertaking genome-wide scans of threespine sticklebacks [11–13, 16, 17], we identified several chromosomal regions that are likely under differential selection in freshwater and marine environments (Figure 2). Comparison of our results with results from whole genome sequencing [12] and RAD sequencing [11] suggests that several of the regions may be the same, thereby also implying that the same candidate genes may be involved. Specifically, there appears to be concordance among the previous and the current study in identifying regions on chromosomes I, IV, VII, IX, XI, XIV, XVI and XX as being involved in freshwater-saltwater adaptation (compare e.g. Figure 2 of the present study with Figure two (a) in [13]).

The identified outlier chromosomal regions harbor a number of candidate genes with functional relationships that are already known to be important for adaptation between freshwater and marine habitats, such as genes affecting bone development, kidney function and osmoregulation (Skeletal Biology: SB; Kidney Function: KF; Osmoregulation: OM ,respectively; see Table 3). We find it interesting that our study reveals two candidate loci (both on chromosome XI; ATP2A2 and ABCA3, see Table 3) putatively implied in ATPase activity, generally associated with salinity tolerance. Other candidate genes related to this ATPase activity have previously been found on chromosome I and in two other regions of chromosome XI [12], and the candidate genes suggested by the current study further emphasize the importance of this physiological trait.

The insulin-like growth factor binding protein 2, IGFBP2 in chromosome I (see Table 3) is another interesting candidate gene observed in the present study that was also suggested as a candidate for freshwater-marine adaptation by Hohenlohe *et al.* [11]. We also note four highly differentiated SNPs in four different chromosomal regions (Table 3); ADAMTS18 in chromosome II, retinol binding protein 4 (RBP4) in chromosome V, lens fiber membrane intrinsic protein 2 (LIM2) in chromosome VII and the retinoic X receptor alpha (RXRA) in chromosome XVI (F_ST_ values ranging from 0.54 to 0.8, Table 2) that could be involved in vision. This could reflect adaptation to different light environments, in the present case between freshwater and marine habitats, as previously observed in other marine organisms [45, 46].

As our SNP resource was specifically designed based on RAD sequencing data, there are a number of candidate genes and chromosomal regions that will inevitably not be represented. First, some candidate genes and SNPs may only be regionally important, as discussed previously. Second, RAD sequencing using the 8-base cutter SbfI obviously provides less resolution than e.g. whole genome sequencing, and there may be regions and candidate genes showing elevated differentiation that have not been detected. Our SNP resource can be regarded as a reduced representation of outlier regions detected by RAD sequencing, which by itself represents a reduced representation of the whole genome. Obviously, the SNP resource can be supplemented by other previously identified candidate genes and markers, and conversely it represents a supplement to the markers and resources already available [10, 12, 13, 17].

## Conclusions

We have constructed a low density SNP array that encompasses both neutral SNPs for background and SNPs representing genomic regions that exhibit differentiation compatible with diversifying selection in freshwater and marine environments. We find this resource to be particularly useful for addressing research questions that require high sample sizes, e.g. several hundreds, which would in most cases not be feasible for whole genome sequencing and RAD sequencing. For instance, this concerns situations where hybrid zone dynamics between freshwater and marine sticklebacks are analyzed along environmental gradients [20]. This may necessitate large sample sizes, e.g. if continuous sampling is conducted in order to identify clinal shifts of allele frequencies [47] or define populations based on neutral or adaptive markers [48]. Also, studies of selection based on detecting allele frequency change using analysis of temporal samples, e.g. taken at different time points within a year [18], may require analysis of many samples and large sample sizes. We find our SNP array to be particularly useful in such situations, as it allows for studies going beyond analyzing EDA and instead targeting multiple genomic regions involved in differential adaptation to freshwater and marine environments. We specifically intend to use the SNP array for testing the hypothesis that gene flow from marine populations overrides selection in freshwater sticklebacks in coastal regions [18]. If this is indeed the case, then this should not only be detectable at the EDA locus but also at other genes involved in adaptive responses, including those represented in our array.

### Availability of supporting data

Sequence reads have been deposited in NCBI’s Sequence Read Archive (Accession number: SAMN0255793).

## Electronic supplementary material

Additional file 1: Table S1: Nucleotide sequences for each SNP position. Fifty nucleotides before and after the targeted position are reported in this table. The two nucleotides corresponding to SNP alleles are presented in brackets. (XLSX 15 KB)

Additional file 2: Table S2: Diversity indices estimated for each locus over 96 individuals from Odder river system. Sample Size (N), observed heterozygosity (Ho), expected heterozygosity (He) and outcomes of tests for Hardy-Weinberg equilibrium (HWE test). *significant at 5% level, **significant at 0.01 level, ***significant at 0.001 level. (XLSX 12 KB)

Additional file 3: **SNP genotypes for 96 SNPs in 96 sticklebacks.** SNP genotype data for 96 SNPs in 96 sticklebacks from the Odder River, Denmark. The data are provided in Genalex 6.5 [27] format. (XLSX 99 kb) (XLSX 100 KB)

## Abbreviations

ABCA3: ATP-binding cassette, sub-family A (ABC1), member 3
ADAMTS18: ADAM metallopeptidase with thrombospondin type 1 motif, 18
ATP2A2: ATPase, Ca++ transporting, cardiac muscle, slow twitch 2
EDA: Ectodysplacin
IGFBP2: Insulin-like growth factor binding protein 2
Indel: Insertion-deletion
KF: Kidney function
LIM2: Lens fiber membrane intrinsic protein 2
OM: Osmoregulation
RAD: Restriction site associated DNA
RBP4: Retinol binding protein 4
RXRA: Retinoic X receptor alpha
SB: Skeletal biology
SNP: Single nucleotide polymorphism.

## References

[1] Stockwell CA, Hendry AP, Kinnison MT (2003). Contemporary evolution meets conservation biology. Trends Ecol Evol.

[2] Kinnison MT, Hendry AP, Stockwell CA (2007). Contemporary evolution meets conservation biology II: Impediments to integration and application. Ecol Res.

[3] Conte GL, Arnegard ME, Peichel CL, Schluter D (2012). The probability of genetic parallelism and convergence in natural populations. P R Soc B.

[4] Miller MR, Brunelli JP, Wheeler PA, Liu SX, Rexroad CE, Palti Y, Doe CQ, Thorgaard GH (2012). A conserved haplotype controls parallel adaptation in geographically distant salmonid populations. Mol Ecol.

[5] Gagnaire PA, Pavey SA, Normandeau E, Bernatchez L (2013). The genetic architecture of reproductive isolation during speciation-with-gene-flow in lake whitefish species pairs asessed by RAD sequencing. Evolution.

[6] Hoekstra HE, Hirschmann RJ, Bundey RA, Insel PA, Crossland JP (2006). A single amino acid mutation contributes to adaptive beach mouse color pattern. Science.

[7] McKinnon JS, Rundle HD (2002). Speciation in nature: the threespine stickleback model systems. Trends Ecol Evol.

[8] Bell MA, Aguirre WE, Buck NJ (2004). Twelve years of contemporary armor evolution in a threespine stickleback population. Evolution.

[9] Le Rouzic A, Østbye K, Klepaker TO, Hansen TF, Bernatchez L, Schluter D, Vollestad LA (2011). Strong and consistent natural selection associated with armour reduction in sticklebacks. Mol Ecol.

[10] Colosimo PF, Hosemann KE, Balabhadra S, Villarreal G, Dickson M, Grimwood J, Schmutz J, Myers RM, Schluter D, Kingsley DM (2005). Widespread parallel evolution in sticklebacks by repeated fixation of ectodysplasin alleles. Science.

[11] Hohenlohe PA, Bassham S, Etter PD, Stiffler N, Johnson EA, Cresko WA (2010). Population genomics of parallel adaptation in threespine stickleback using sequenced RAD tags. PLoS Genet.

[12] Jones FC, Chan YF, Schmutz J, Grimwood J, Brady SD, Southwick AM, Absher DM, Myers RM, Reimchen TE, Deagle BE, Schluter D, Kingsley DM (2012). A genome-wide SNP genotyping array reveals patterns of global and repeated species-pair divergence in sticklebacks. Curr Biol.

[13] Jones FC, Grabherr MG, Chan YF, Russell P, Mauceli E, Johnson J, Swofford R, Pirun M, Zody MC, White S, Birney E, Searle S, Schmutz J, Grimwood J, Dickson MC, Myers RM, Miller CT, Summers BR, Knecht AK, Brady SD, Zhang HL, Pollen AA, Howes T, Amemiya C, Lander ES, Di Palma S, Lindblad-Toh K, Kingsley DM (2012). The genomic basis of adaptive evolution in threespine sticklebacks. Nature.

[14] Mäkinen HS, Cano M, Merila J (2008). Identifying footprints of directional and balancing selection in marine and freshwater three-spined stickleback (*Gasterosteus aculeatus*) populations. Mol Ecol.

[15] Rogers SM (2012). Mapping the genomic architecture of ecological speciation in the wild: does linkage disequilibrium hold the key?. Mol Ecol.

[16] DeFaveri J, Shikano T, Shimada Y, Goto A, Merila J (2011). Global analysis of genes involved in freshwater adaptation in threespine sticklebacks (*Gasterosteus aculeatus*). Evolution.

[17] Shimada Y, Shikano T, Merila J (2011). A high incidence of selection on physiologically important genes in the three-spined stickleback, *Gasterosteus aculeatus*. Mol Biol Evol.

[18] Raeymaekers JA, Konijnendijk N, Larmuseau MH, Hellemans B, De Meester L, Volckaert FA (2014). A gene with major phenotypic effects as a target for selection vs. homogenizing gene flow. Mol Ecol.

[19] McCairns RJS, Bernatchez L (2008). Landscape genetic analyses reveal cryptic population structure and putative selection gradients in a large-scale estuarine environment. Mol Ecol.

[20] Jones FC, Brown C, Pemberton JM, Braithwaite VA (2006). Reproductive isolation in a threespine stickleback hybrid zone. J Evol Biol.

[21] Baird NA, Etter PD, Atwood TS, Currey MC, Shiver AL, Lewis ZA, Selker EU, Cresko WA, Johnson EA (2008). Rapid SNP discovery and genetic mapping using sequenced RAD markers. PLoS One.

[22] Pujolar JM, Jacobsen MW, Frydenberg J, Als TD, Larsen PF, Maes GE, Zane L, Jian JB, Cheng L, Hansen MM (2013). A resource of genome-wide single-nucleotide polymorphisms generated by RAD tag sequencing in the critically endangered European eel. Mol Ecol Resour.

[23] Langmead B, Trapnell C, Pop M, Salzberg SL (2009). Ultrafast and memory-efficient alignment of short DNA sequences to the human genome. Genome Biol.

[24] Catchen J, Hohenlohe PA, Bassham S, Amores A, Cresko WA (2013). Stacks: an analysis tool set for population genomics. Mol Ecol.

[25] Hoekstra HE (2012). Genomics: Stickleback is the catch of the day. Nature.

[26] Seeb JE, Pascal CE, Ramakrishnan R, Seeb LW (2009). SNP genotyping by the 5′-nuclease reaction: advances in high-throughput genotyping with nonmodel organisms. Methods Mol Biol.

[27] Peakall R, Smouse PE (2012). GenAlEx 6.5: genetic analysis in Excel. Population genetic software for teaching and research-an update. Bioinformatics.

[28] Benjamini Y, Yekutieli D (2001). The control of the false discovery rate in multiple testing under dependency. Ann Stat.

[29] Ellegren H (2014). Genome sequencing and population genomics in non-model organisms. Trends Ecol Evol.

[30] Allendorf FW, Hohenlohe PA, Luikart G (2010). Genomics and the future of conservation genetics. Nat Rev Genet.

[31] Davey JW, Hohenlohe PA, Etter PD, Boone JQ, Catchen JM, Blaxter LM (2011). Genome-wide genetic marker discovery and genotyping using next-generation sequencing. Nat Rev Genet.

[32] Renaut S, Grassa CJ, Yeaman S, Moyers BT, Lai Z, Kane NC, Bowers JE, Burke JM, Rieseberg LH (2013). Genomic islands of divergence are not affected by geography of speciation in sunflowers. Nat Commun.

[33] Moura AE, Janse van Rensburg C, Pilot M, Tehrani A, Best PB, Thornton M, Plon S, de Bruyn PJ, Worley KC, Gibbs RA, Dahlheim ME, Hoelzel AR (2014). Killer whale nuclear genome and mtDNA reveal widespread population bottleneck during the last glacial maximum. Mol Biol Evol.

[34] Hess JE, Campbell NR, Close DA, Docker MF, Narum SR (2013). Population genomics of Pacific lamprey: adaptive variation in a highly dispersive species. Mol Ecol.

[35] Hess JE, Campbell NR, Docker MF, Baker C, Jackson A, Lampman R, McIlraith B, Moser ML, Statler DP, Young WP, Wildbill AJ, Narum SR (2014). Use of genotyping by sequencing data to develop a high-throughput and multifunctional SNP panel for conservation applications in Pacific lamprey. Mol Ecol Resour.

[36] Pujolar JM, Jacobsen MW, Als TD, Frydenberg J, Magnussen E, Jonsson B, Jiang X, Cheng L, Bekkevold D, Maes GE, Bernatchez L, Hansen MM (2014). Assessing patterns of hybridization between North Atlantic eels using diagnostic single-nucleotide polymorphisms. Heredity.

[37] Albrechtsen A, Nielsen FC, Nielsen R (2010). Ascertainment biases in SNP chips affect measures of population divergence. Mol Biol Evol.

[38] Rosenblum EB, Novembre J (2007). Ascertainment bias in spatially structured populations: A case study in the eastern fence lizard. J Hered.

[39] DeFaveri J, Viitaniemi H, Leder E, Merila J (2013). Characterizing genic and nongenic molecular markers: comparison of microsatellites and SNPs. Mol Ecol Res.

[40] Morin PA, Luikart G, Wayne RK (2004). SNPs in ecology, evolution and conservation. Trends Ecol Evol.

[41] Glover KA, Hansen MM, Lien S, Als TD, Hoyheim B, Skaala O (2010). A comparison of SNP and STR loci for delineating population structure and performing individual genetic assignment. BMC Genet.

[42] Chorev M, Carmel L (2012). The function of introns. Front Genet.

[43] Hebert FO, Renaut S, Bernatchez L (2013). Targeted sequence capture and resequencing implies a predominant role of regulatory regions in the divergence of a sympatric lake whitefish species pair (*Coregonus clupeaformis*). Mol Ecol.

[44] The ENCODE Project Consortium (2012). An integrated encyclopedia of DNA elements in the human genome. Nature.

[45] Audzijonyte A, Pahlberg J, Viljanen M, Donner K, Vainola R (2012). Opsin gene sequence variation across phylogenetic and population histories in Mysis (Crustacea: Mysida) does not match current light environments or visual-pigment absorbance spectra. Mol Ecol.

[46] Larmuseau MHD, Raeymaekers JAM, Ruddick KG, Van Houdt JKJ, Volckaert FAM (2009). To see in different seas: spatial variation in the rhodopsin gene of the sand goby (Pomatoschistus minutus). Mol Ecol.

[47] Derryberry EP, Derryberry GE, Maley JM, Brumfield RT (2014). hzar: hybrid zone analysis using an R software package. Mol Ecol Resour.

[48] Guillot G, Mortier F, Estoup A (2005). GENELAND: a computer package for landscape genetics. Mol Ecol Notes.

